# Obtainment and Characterization of Metal-Coated Polyethylene Granules as a Basis for the Development of Heat Storage Systems

**DOI:** 10.3390/polym14010218

**Published:** 2022-01-05

**Authors:** Volodymyr Moravskyi, Anastasiia Kucherenko, Marta Kuznetsova, Ludmila Dulebova, Emil Spišák

**Affiliations:** 1 Department of Chemical Technology of Plastics Processing, Lviv Polytechnic National University, 12, Bandera Str., 79013 Lviv, Ukraine; vmoravsky@gmail.com (V.M.); anastasiyakucherenko05@gmail.com (A.K.); 2 Department of Heat Engineering and Thermal and Nuclear Power Plants, Lviv Polytechnic National University, 12, Bandera Str., 79013 Lviv, Ukraine; kuznetsovam83@gmail.com; 3 Department of Technologies, Materials and Computer Aided Production, Faculty of Mechanical Engineering, Technical University of Košice, 74 Mäsiarska, 04001 Košice, Slovakia; emil.spisak@tuke.sk

**Keywords:** polyethylene, copper, heat accumulator, metallization, chemical reduction, metal-filled polymer composite

## Abstract

The research studied the feasibility of using copper-coated polyethylene granules as a basis for creating efficient heat storage systems. A technology for imparting catalytic properties to a polymer surface by the joint processing of polymer granules and an activator metal in a ball mill with their subsequent metallization in a chemical reducing solution is proposed. The efficiency of copper-coating a polyethylene surface is shown to be largely determined by the activation stage and the assumption regarding the mechanism of interaction of the activator metal with the polymer surface is made. To obtain different amounts of metal on the polyethylene granules, it is proposed that the method of remetallization is used. It was established that the rate of copper ion reduction depends on the number of previous coatings and is determined by the area of interaction of the metal-coated granules with the chemical reducing solution. The obtained metal-coated polyethylene granules were characterized in terms of the viability of using it as a phase transition material for a heat storage system. Using the developed installation that simulates the heat accumulator operation, it was shown that the efficiency of using metal-coated polyethylene granules to create heat storage systems is higher. The copper coating deposited on the polyethylene granules was studied using scanning electron microscopy and X-ray diffraction analysis.

## 1. Introduction

Polymers, as a matrix for creating composite materials, offer significant prospects. This is due to the possibility to use various fillers, as well as the high manufacturability of the polymer matrix, which makes it possible to process such composites and obtain products from them by high-performance methods. In addition, using a remarkably wide range of polymers on the market, one can control the properties of the final product, which, along with the use of different fillers, makes it possible to obtain materials with the desired properties [1,2,3,4,5,6,7,8,9,10].

Metal-filled materials make up a separate group of polymer composites. Such composites combine the characteristics of materials that differ significantly in their properties. For example, metals and polymers are materials with substantially different electrical, heat conduction and magnetic properties. Thus, the combination of metals and polymers in one composite makes it possible to obtain new materials with unique properties and a wide range of prospective applications in medicine, construction, aviation, etc. [11,12,13].

There are many technologies for obtaining metal-filled polymer composites. The most common are methods that involve mixing composite components in the viscous-flow state of the polymer [14,15]. Other promising methods include metal reduction in the polymer network, adding metal during polymer synthesis and sol-gel technologies [16,17,18,19,20,21]. Metal-filled polymer products can be obtained using 3D printing technologies [22,23]. Metal alloys, in particular eutectic mixtures, are also used to create metal-filled polymer composites. The specific feature of obtaining such composites is the use of temperature modes at which both the polymer and the metal are in a molten state. This approach ensures high values of conductivity of the composite at low filling degrees, especially when combining fusible systems and traditional conductive fillers [24,25,26,27,28]. These different technologies are aimed at obtaining materials with uniform distribution of metal in the polymer matrix, as well as obtaining metal filler of a particular dimension, which has a strong impact on the properties of the resulting composite [11,29,30,31].

The properties and characteristics of the obtained composites determine possible areas of their application. Conductive and magnetic properties of metal-filled composites can be used to create electromagnetic shield materials [24,32,33,34]. A separate area of application of metal-filled composites relies on their high thermal conductivity [35,36,37,38]. Such composites can be successfully used in heat transfer systems for which the main factor is the ratio of the efficiency to the cost. This combination becomes possible due to maintaining the high processability of the polymer matrix and an increased thermal conductivity provided by adding a metal filler.

Given the improved thermal conductivity of metal-filled polymer composites, alternative energy is a promising area for their use, namely as highly efficient heat storage systems. The problem of accumulation, storage and subsequent use of energy is typical for both conventional and alternative power engineering [39,40]. The introduction of reduced night electricity tariffs aims to equalize daily electricity consumption by stimulating the end consumer to use energy at night more intensively. One of the most energy-intensive systems whose operation can rely on using the night tariff is the heating system. The introduction of heating systems with energy storage will solve several problems associated with the inconsistency in energy production and consumption schedules in both conventional and alternative power engineering. Out of the different types of heat storage systems, those using phase transition heat are considered the most promising. Their significant advantage is the high-density energy storage in a narrow temperature range [39,40,41,42,43].

Crystalline polymers can be used as a basis for creating phase transition heat storage systems [44]. However, polymers have poor thermal conductivity, which imposes certain limitations on the development of highly efficient heat storage systems on their basis. Cooling of the surface layers of the polymer in contact with the heat transfer surface significantly reduces heat extraction from the inner heated layers, which reduces the efficiency of the heat storage system. This disadvantage requires the development of new materials that will allow rapid and uniform heating of the polymer (charge) and efficient extraction of the accumulated heat (discharge).

There are many options to increase the efficiency of heat storage/heat transfer by heat accumulators. The thermal conductivity of phase transition materials can be increased by using porous media [45,46], various designs of heat exchangers with additional elements on them [47,48] and nanosized additives [49,50,51]. Moreover, it is noted that the form of the filler used to increase thermal conductivity also has an effect on the charge time of the phase transition material, and elongated fillers offer higher efficiency [52].

Thus, it is necessary to develop polymer heat-accumulating material that will have an increased thermal conductivity (due to the introduction of metal filler) and high values of stored energy density (due to the phase transition of crystalline polymers). The article discusses an experimental study of the feasibility of using commercially available polyethylene granules as a phase transition material. The technology for increasing the efficiency of using phase transition material in heat accumulation systems due to the metallization of its surface is proposed. The developed material was evaluated by using an experimental setup simulating the heat accumulator operation. The efficiency of using the phase transition material depending on the metal content was evaluated and the influence of the metal on the efficiency of using the phase transition material was confirmed.

## 2. Materials and Methods

The developed technique, which includes the activation of the surface of the granules and their subsequent metallization in chemical reducing solutions, was used to obtain metallized polyethylene granules (Liten PL-10 (Unipetrol, Litvínov, Czech Republic)) [53,54,55,56,57].

### 2.1. Activation of the Polymer Surface

Fine-grained zinc powder (ПЦ-2 grade) was used to activate the surface of the polyethylene granules. The activation consists of the joint processing of polymer granules and an activator metal in a ball mill filled with ceramic grinding bodies. This treatment ensures the fixation of zinc particles on the surface of the polymer granules and imparts catalytic properties to it in the processes of chemical deposition of copper from solutions. The activation of the polyethylene granules was performed in a four-liter laboratory ball mill with ceramic cylindrical grinding bodies for 1 h at a rotation speed of 125 rpm. A total of 100 g polymer and 5 g fine-grained zinc powder were loaded into the mill; the weight of the grinding bodies was 1.5 [58]. The obtained activated polyethylene granules were washed with water to remove zinc particles that failed to fix on the surface of the granules.

### 2.2. Metallization of Activated Polyethylene Granules and Study of Its Kinetics

The metallization of the activated polyethylene granules was performed in a formalin-stabilized (365 mmol/L) chemical reducing solution of the following composition: 50 mmol/L CuSO_4_∙5H_2_O (pure for analysis), 67 mmol/L EDTA-Na_2_ (C_10_H_14_N_2_Na_2_O_8_∙2H_2_O) (pure for analysis), 375 mmol/L NaOH (pure). This composition of the chemical reducing solution, on condition of a complete reduction of copper ions (0.05 mol/L), makes it possible to obtain a 3.1 g copper coating according to the scheme of copper ion reduction with formaldehyde. In the studies on the kinetics of copper ion reduction, the activated polyethylene weight was 20 g with the chemical reducing solution volume being 200 mL. This ratio between the activated polymer weight and the chemical reducing solution volume was also preserved for obtaining copper-coated polyethylene granules in the other studies.

The kinetic regularities of copper reduction on the zinc-activated polyethylene surface were studied by the volumetric method. This method is based on measuring the volume of gas released as a result of copper reduction. According to [59], in chemical metallization solutions with the complexing agent, EDTA-Na_2_, the reduction of one mole of copper releases one mole of hydrogen. This feature allows obtaining the dependence of the change in the copper concentration in the chemical metallization solution over time, i.e., the kinetic curves of copper reduction. The kinetic curves of copper reduction presented in the article are the average result of several studies, the relative measurement error of which does not exceed 5%.

The measurement device consisted of a reaction vessel for metallization, a gas burette and a balancing bulb. The gas volume of the burette was connected to the reaction vessel and the water volume to the balancing bulb. The amount of hydrogen released during copper reduction was determined by the volume of the water displaced from the burette.

The metallization efficiency was calculated as the ratio of the weight of copper deposited on the polymer surface to the theoretical weight of copper, which can be obtained on condition that it is completely reduced from the solution. To this end, the copper granules were weighed to the nearest 0.00005 g, treated with 50% nitric acid for 5 min, washed, dried to a constant weight and weighed again. The percentage of metal on the metallized granules was calculated from the difference between the granules weight before and after etching in the acid.

The applied technique of remetallization includes performance of the first coating of the zinc-activated polyethylene granules with copper in the chemical reducing solution of the above-indicated composition. For the second metallization, the copper-coated polyethylene granules obtained during the first metallization and the chemical reducing solution of the above-indicated composition were used. The polyethylene granules coated with copper twice were used as raw materials for the third metallization as well as the chemical reducing solution of the same composition. The polyethylene granules coated with copper three times were used as raw materials for the fourth metallization plus the chemical reducing solution of the same composition; the fifth metallization used the polyethylene granules coated with copper four times and the chemical reducing solution of the same composition, and so on. The use of the remetallization technique allows increasing the metal content and thickness of the copper coating on the surface of polyethylene granules.

### 2.3. Test Methods

Microscopic examinations were performed using a scanning electronic microscope Zeiss EVO-40XVP (ZEISS, Jena, Germany) supplied with the systems of energy dispersive microanalysis and inverted electron diffraction. 

The crystal structure of the samples was analyzed by X-ray diffractometry (XRD), for which a DRON-4-07 X-ray Diffractometer (Bourevestnik JSC, Saint Petersburg, Russia) was used. Irradiating lamps with a copper anode and Ni-filter were used. The studies were carried out in the range of 2Θ from 4 to 360°.

The melt flow index (MFI) was obtained on the device IIRT-AM (ASMA-Pribor, Svitlovodsk, Ukraine) at a temperature of 190 °C and load of 5 kg.

### 2.4. Experimental Setup Simulating the Heat Accumulator Operation

**Figure 1:**
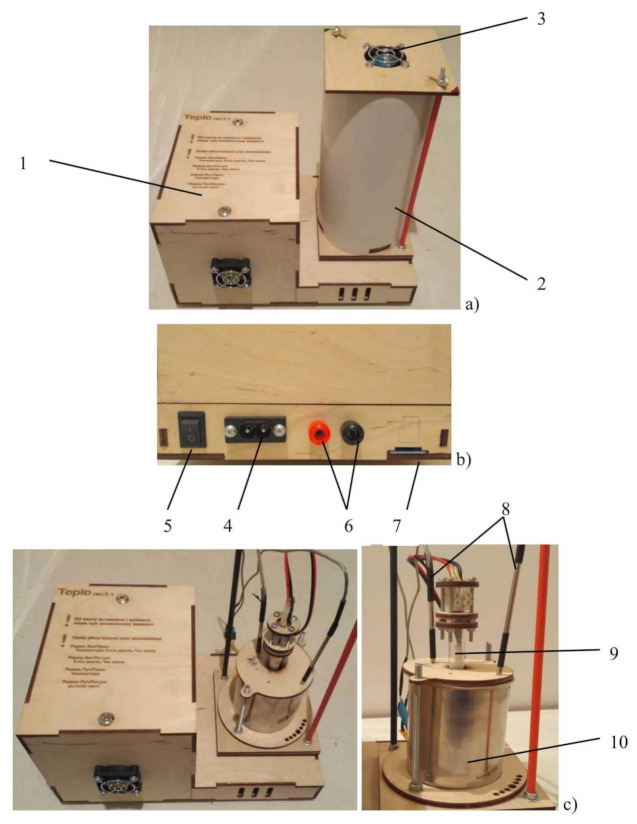
The experimental setup for simulating the heat accumulator operation. (**a**) general view, (**b**) connection panel, (**c**) measuring cell. 1—control unit, 2—insulating cover, 3—cooling fan, 4—power supply, 5—switch, 6—terminals for connecting the laboratory power supply unit, 7—SD card slot, 8—thermocouples, 9—heater, 10—container with phase transition substance.

In order to evaluate the efficiency of using the obtained metallized polyethylene as a basis for the creation of phase transition heat storage systems, an experimental setup was developed that simulates the operation of the heat accumulator. The main units of the setup are the control unit and the measuring cell (Figure 1).

**Figure 2:**
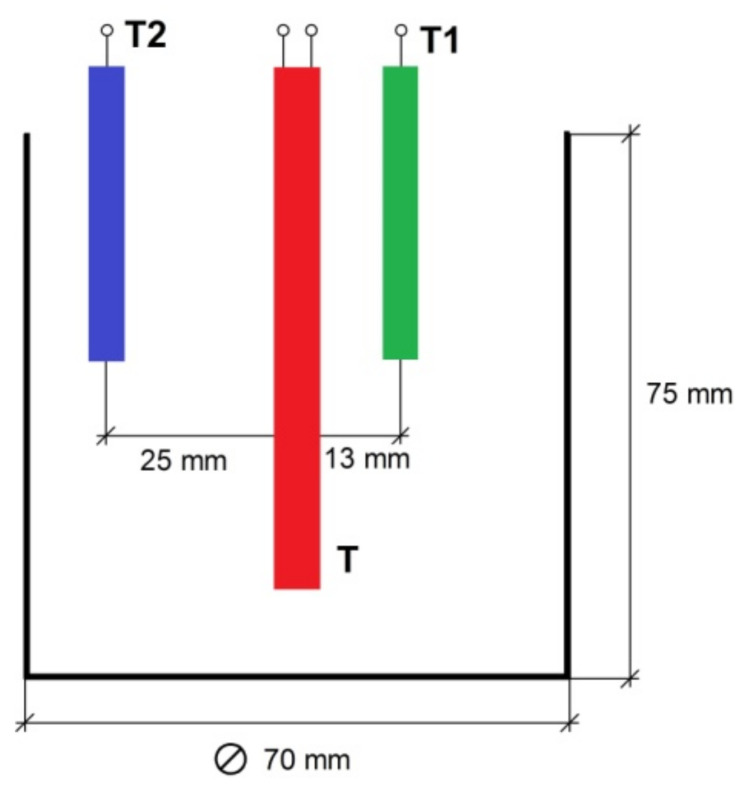
The main dimensions of the measuring cell.

The measuring cell is located inside the insulating cover, in the bottom part of which slots are made to ensure air circulation during the discharge of the accumulator. To intensify air circulation, there is a fan in the upper part of the insulating cover. The measuring cell consists of a metal container comprising the phase transition material, a heater and two thermocouples for measuring the change of the material temperature during the charge and discharge of the accumulator. The dimensions of the measuring cell and the relative location of the thermocouples and the heater are shown in Figure 2.

The experimental setup can work in the manual and automatic modes and is based on the ATmega2560 microcontroller. A piece of software supporting the Android operating system has been developed to control the operation of the setup. It makes it possible to carry out a study in one of the modes with a graphical display of the current values of all the parameters and their saving for further analysis.

**Figure 3:**
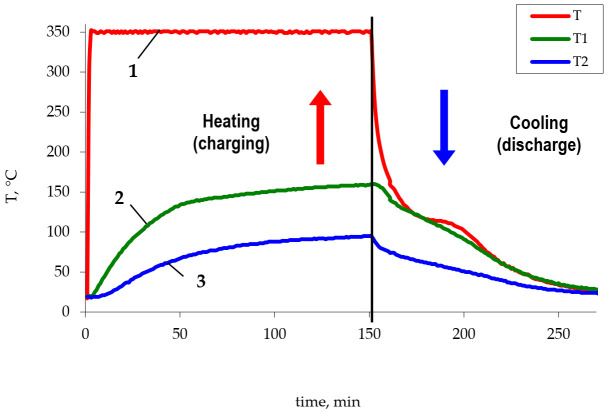
The change of the Liten PL-10 polyethylene temperature. 1—heater temperature, 2—temperature of thermocouple 1, 3—temperature of thermocouple 2.

The study of the heat accumulator operation consisted of measuring changes in the temperature of the phase transition material (polyethylene and metallized polyethylene) over time in the charge and discharge modes (Figure 3). The heater temperature was the same in all cases. The obtained dependences were used to determine the temperature of thermocouples at fixed time intervals, based on which the efficiency of the metallized polyethylene use was evaluated.

## 3. Results and Discussion

### 3.1. Activation of Polyethylene Granules

**Figure 4:**
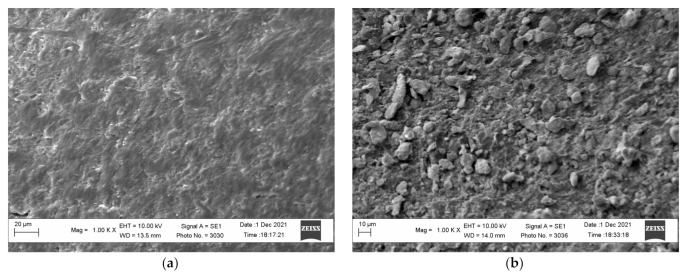
The micrograph of the surface of the initial polyethylene granules (**a**) and zinc-activated surface of the polyethylene granules (**b**).

**Figure 5:**
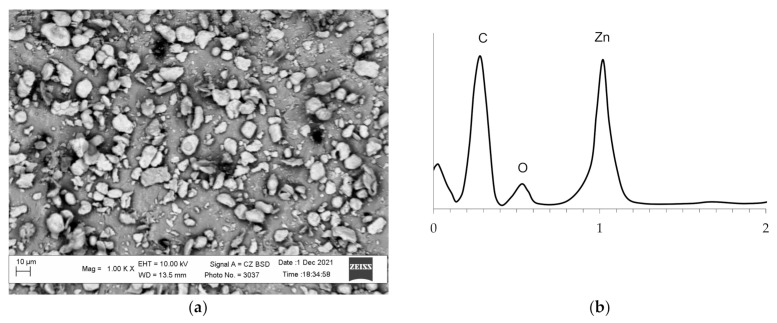
The images of the zinc-activated polyethylene surface obtained in the contrast mode by the mean atomic number (**a**) and energy dispersive analysis of the surface (**b**).

The activation of the polymer surface by the developed technology allows obtaining a raw polymer material, the surface of which contains particles of the catalytically active metal. The study of the surface of the obtained polyethylene granules and the activated surface performed on the scanning electronic microscope indicate that the polyethylene surface changed essentially and was saturated with zinc particles (Figure 4). The presence of zinc particles on the surface of the activated polyethylene granules is corroborated by the results of analysis of the images obtained in the contrast mode by the mean atomic number and by energy dispersive analysis (Figure 5). In this case, the zinc particles are brighter and can be clearly identified in the micrographs. The analysis of the micrographs suggests that the zinc particles are evenly distributed on the surface and probably interact with it.

**Figure 6:**
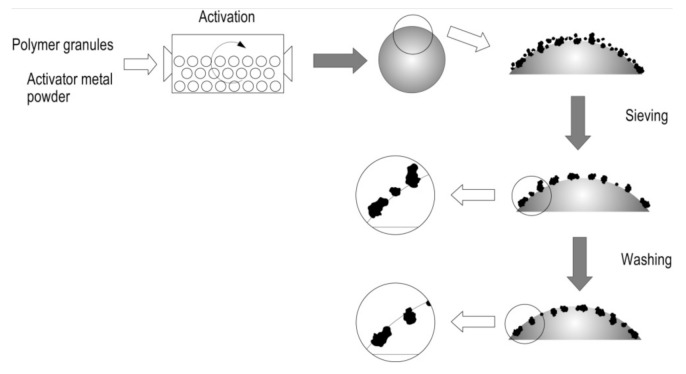
The scheme of activation of the polyethylene granules and interaction of zinc particles with their surface.

The interaction of zinc particles with the polymer surface is caused by the impact action created by the presence of the ceramic grinding bodies in the ball mill. As the mill rotates, the polyethylene granules get coated with fine zinc particles, which at the initial stage interact due to electrostatic forces. Then, as the mill continues to rotate, the impact action of the grinding bodies on the polymer surface occurs, as a result of which the zinc particles penetrate into the polymer surface (Figure 6). Such penetration is possible owing to the higher hardness of zinc in comparison with polyethylene. The amount of zinc fixed on the polymer surface depends on the rate and duration of activation, the ratio between the polymer and the metal activator, as well as the degree of ball mill loading [58].

**Figure 7:**
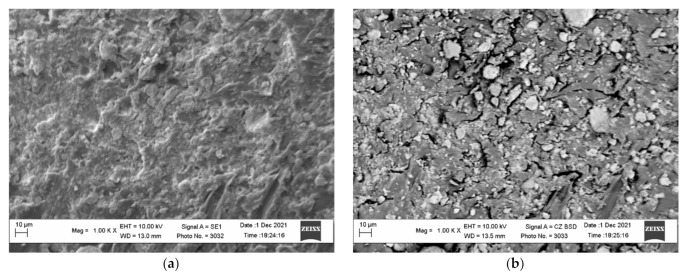
The micrographs of the surface of the activated and washed polyethylene granules (**a**) and the images of the surface obtained in the contrast mode by the mean atomic number (**b**).

It should be noted that the activation of the polymer surface is extremely important for obtaining metallized raw materials of the required quality. Immediately after the activation in the ball mill, the activated granules are sieved and washed to remove loose zinc particles from the polymer surface. The comparison of the images in Figure 4b and Figure 7 shows that the washed activated polymer surface loses a certain quantity of zinc particles. However, there are still some zinc particles, which is revealed by the surface images obtained in the contrast mode by the mean atomic number.

**Figure 8:**
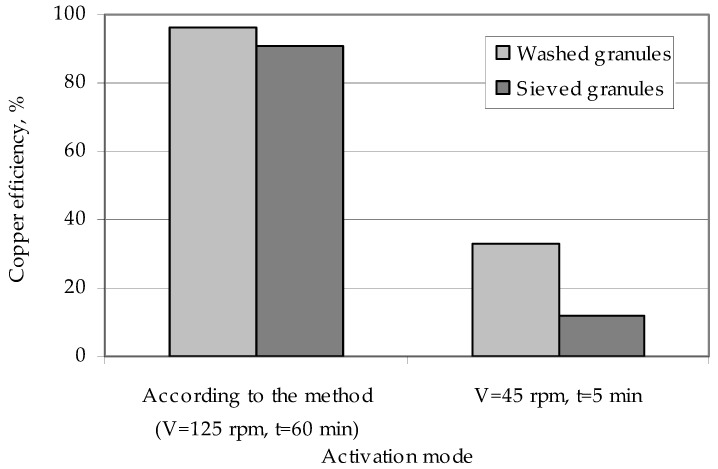
The influence of the method of polyethylene granules preparation on the efficiency of their coating with copper.

This washing increases the efficiency of the copper deposition on the polymer surface (Figure 8). The effect of weakly fixed zinc particles on the efficiency of metallization can be explained by them being washed in a chemical reducing solution during copper coating of the granules. Flushing of a number of zinc particles means that part of the copper is reduced not on the activated surface of the granules, but in the bulk of the solution on the washed zinc particles. This metal, reduced in the bulk of the solution, forms a precipitate and reduces the efficiency of copper deposition on the granules. Studies of activated polyethylene granules, which are obtained in the modes providing for low values of interaction of zinc particles with the polymer surface (a low speed of the ball mill and low activation time), show that in this case there is almost complete washing of the activator metal from the surface of the polyethylene granules and a reduction in copper ions in the bulk of the solution [55], not on the surface of the granules.

### 3.2. Metallization of Polyethylene Granules

The presence of zinc on the surface of the polyethylene granules provides the possibility of metallization in chemical reducing solutions, the main component of which is copper sulphate. The use of zinc activates the process of copper deposition from the solution and the subsequent formation of copper coating is an autocatalytic process that occurs at a high rate. In the proposed technology, one liter of chemical reducing solution theoretically allows obtaining a copper coating weighing 3 g, which is evenly distributed on the surface of all the granules. Thus, to obtain a metal-coated raw polymer material with a target metal content, it is necessary either to change the amount of the activated polymer loaded into the chemical reducing solution or to use the remetallization method. In this study, the method of remetallization was used to obtain metal-coated polyethylene granules with various copper contents. Such multiple metallization becomes possible owing to the fact that the preformed copper layer has a catalytic activity in chemical reducing solutions [59] and activates the process of copper deposition.

**Figure 9:**
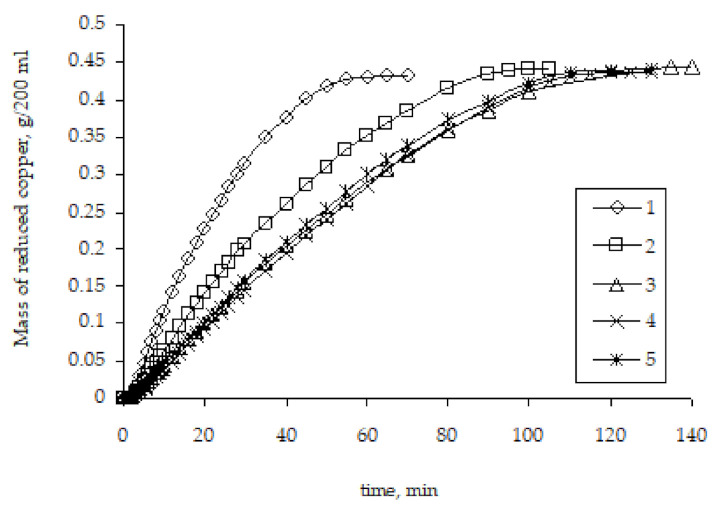
The influence of the number of metallizations on the kinetic curves of copper ion reduction on the surface of polyethylene granules (the number means the number of performed metallizations).

Studies of the kinetics of copper ion reduction as a result of the remetallization of pre-metallized polyethylene granules established that the rate of copper ion reduction significantly depends on the metallization history, namely the number of metallization cycles (Figure 9).

The highest rate of copper ion reduction is observed for the original granules of activated polyethylene. For such granules, the metal activator of the start of the copper ion reduction in the solution is zinc. In the case of repeated metallizations, when previously metallized polyethylene granules are used, the rate of copper ion reduction is lower. This pattern carries on to the third metallization. The rate of subsequent metallizations practically does not depend on the history of the granules used for metallization.

**Figure 10:**
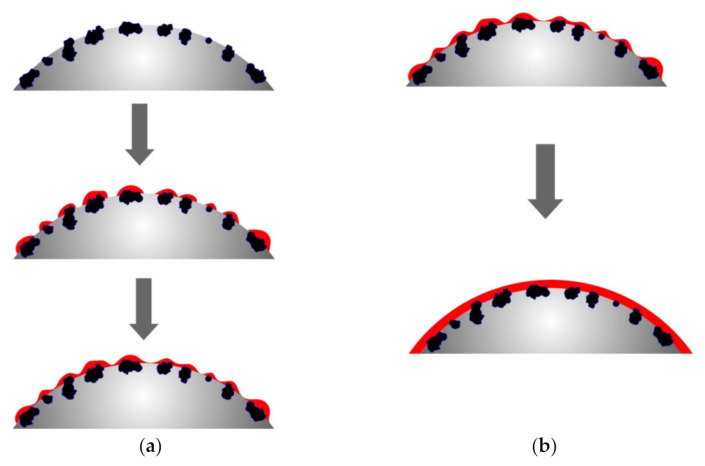
The scheme of decreasing the area during the metallization of polyethylene granules. (**a**) metallization of zinc-activated polyethylene granules; (**b**) remetallization of polyethylene granules.

The different rates of copper ion reduction depending on the number of previous metallizations of polyethylene granules can be explained by the different areas with which the chemical reducing solution comes into contact, as well as the different activity of the metal that catalyzes the copper ion reduction. Obviously, metallic copper formed as a result of previous metallizations has lower catalytic properties compared to zinc, which causes slightly lower copper ion reduction rates. However, the main factor that determines the rate of copper ion reduction, in our opinion, is the area of the surface on which the metal is deposited. As mentioned above, the activated polyethylene granules contain zinc particles on the surface, which form the copper ion reduction centers. The formation of the metal coating starts at such centers with their subsequent fusion and formation of a continuous copper layer. Accordingly, remetallization will smooth the microroughness of the copper layer surface, which will result in the decreased contact area of the surface of the granules with the chemical deposition solution and, accordingly, the decreased rate of copper ion reduction (Figure 10). The proposed explanation of the decreased metallization rate in the case of copper re-coating can be considered the most viable.

Another factor that was considered as an explanation of the metallization rate was the Zn^2+^ ions’ presence in the chemical deposition solution.

**Figure 11:**
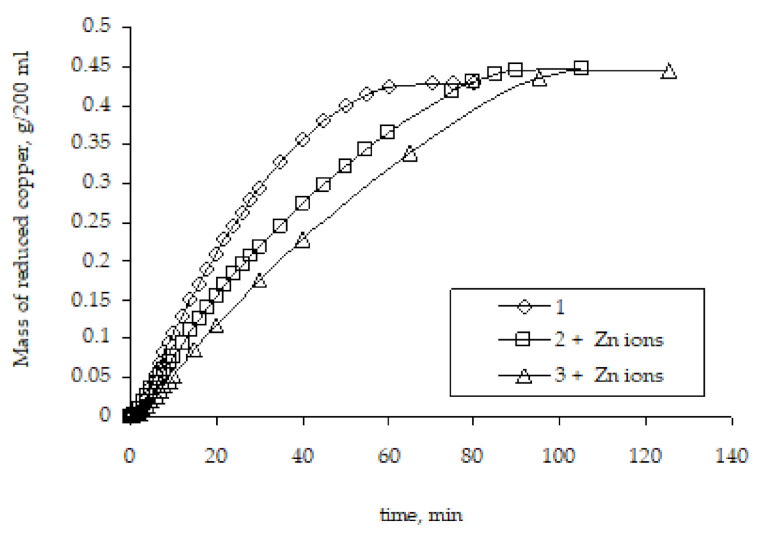
The influence of Zn^2+^ ions presence on the kinetic curves of copper ion reduction on the surface of polyethylene granules (the number means the number of performed metallizations; + Zn ions means the introduction of zinc ions into the chemical reducing solution).

It can be assumed that the presence of zinc ions, which are formed during the copper ion reduction, may have some effect on the metallization rate, namely accelerate it. Since these ions are present in the solution only at the stage of metallization of the initial zinc-activated polyethylene granules and are absent in repeated metallizations, this assumption seems logical. However, studies of the remetallization of the copper-coated polyethylene granules have shown that the presence of Zn^2+^ ions in the chemical reducing solution does not affect the regularities of copper reduction. The rate of remetallization is lower compared to the rate of the metallization of the original activated granules (Figure 11). Hence, the comparison of the remetallization results obtained in the absence of zinc ions (Figure 9) and in their presence in the chemical reducing solution (Figure 11) makes it possible to conclude that the presence of zinc ions has no effect whatsoever on the copper ion reduction rate, and the determining factor is the difference in the surface area in contact with the chemical reducing solution.

**Figure 12:**
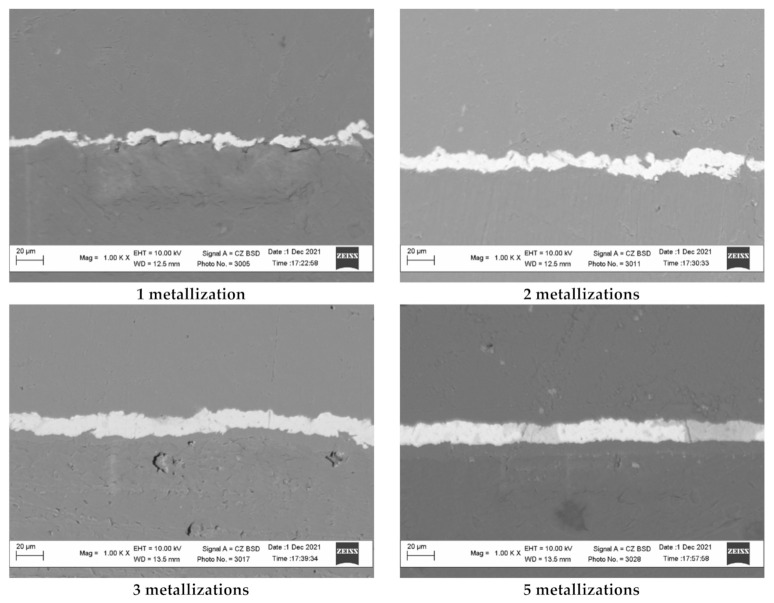
The transverse section of the copper coating obtained on the polyethylene granules as a result of different numbers of remetallizations.

The above assumptions can be confirmed by the study of the surface of copper-coated polyethylene granules after different numbers of metallization cycles using scanning electron microscopy (Figure 12). The relief of the obtained copper coating was studied using the image of the transverse section of the copper-coated granules. The analysis of the images obtained at the same magnification shows that the copper surface obtained after one and two remetallizations exhibits a more developed surface due to a larger microroughness. As the number of remetallizations increase, the surface smooths out and, accordingly, the surface area decreases.

**Figure 13:**
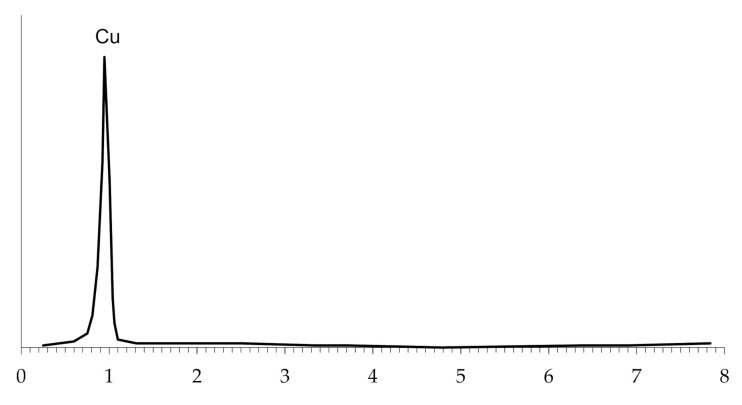
The energy dispersive analysis of the surface of copper-plated polyethylene granules.

The microscopic study of the copper-coated polyethylene granules, regardless of the number of metallizations, show that a dense and continuous copper coating forms on the surface of the granules, which was confirmed by energy dispersive analysis (Figure 13).

**Figure 14:**
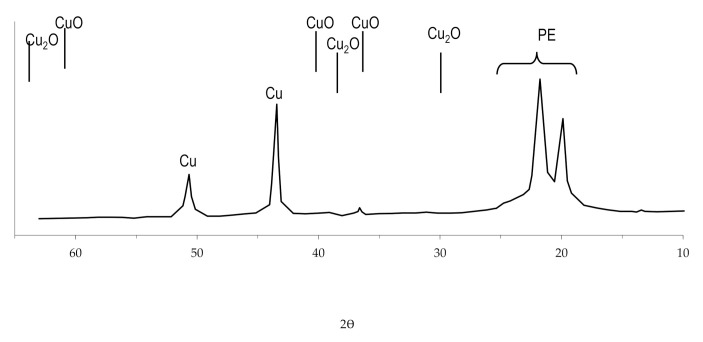
The radiograph of copper coating obtained by chemical reduction of copper ions from solution with the complexing agent EDTA-Na_2_.

The study of the obtained copper coating using X-ray diffraction analysis revealed that there are no oxides in the structure of the coating (Figure 14). This feature of copper coatings obtained by the chemical reduction of copper ions in solutions with the complexing agent EDTA-Na_2_ was also noted by other researchers [59] and was investigated when obtaining copper coating on other polymers, using the developed technology [54,57].

**Figure 15:**
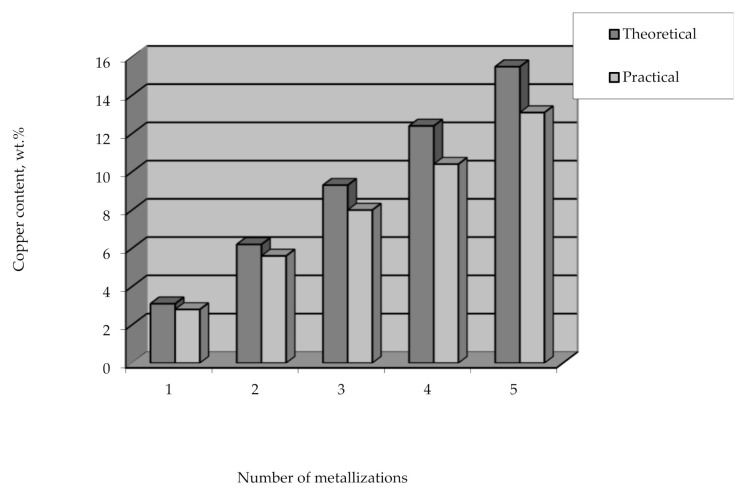
The content of copper deposited on polyethylene granules vs the number of metallizations.

Applying the method of the remetallization of polyethylene granules allows obtaining raw polymer materials with various metal contents. In this case, the required amount of metal can be easily and clearly controlled by the number of metallizations. It should also be noted that the efficiency of copper coating is high and amounts to over 90% of the theoretical amount of copper that can be deposited from the solution (Figure 15).

### 3.3. Efficiency of Using Metallized Polyethylene as a Basis for the Creation of Phase Transition Heat Storage Systems

The obtained metallized polyethylene granules are considered as a basis for the creation of heat storage systems. Polyethylene is a highly crystalline polymer, which makes it suitable for developing highly efficient heat storage systems, in which the presence of metal particles in the bulk of the polymer will increase thermal conductivity. According to the developed technology, the metal is introduced in the form of a metal coating deposited directly on the phase transition material (polyethylene). This method will ensure the simple adding of metal into the heat storage system while forming a homogeneous system with a uniform distribution of the metal in the bulk of polyethylene.

**Figure 16:**
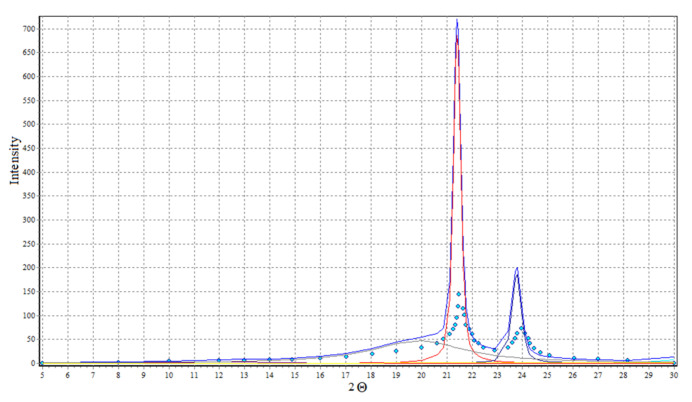
X-ray diffraction curves of polyethylene.

Analysis of diffraction curves of X-ray (Figure 16) with WAXSFIT [60] software product application allows the estimation of the crystallinity degree of used polyethylene at 64%.

**Figure 17:**
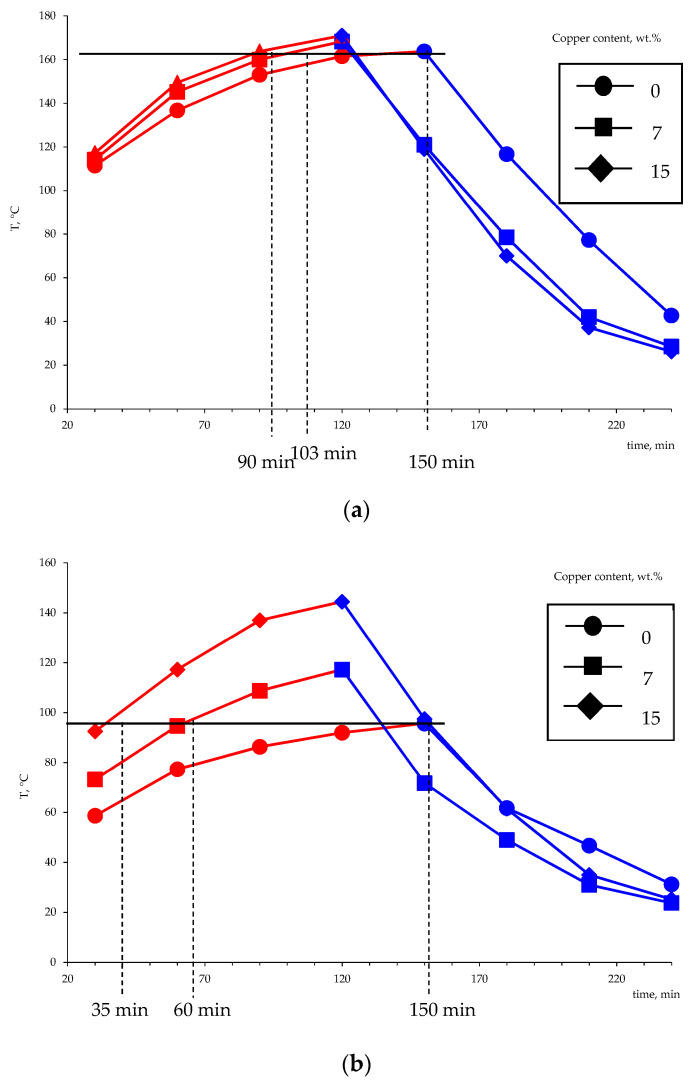
The variation of the temperature of the heat-accumulating material based on the Liten PL-10 polyethylene in the charging-discharging modes as measured by the thermocouples T1 (**a**) and T2 (**b**) (according to the diagram in Figure 3).

Using the developed setup simulating the heat accumulator operation, the influence of the metal amount on the performance characteristics of the setup in the charge and discharge modes was studied. The experiment consisted of measuring changes in the temperature of the phase transition material over time. Several charge–discharge cycles were performed to compact the material and obtain a monolithic block. The obtained dependencies of the mean temperature of the thermocouples T1 and T2 (five charging–discharging cycles) during the heating and cooling are shown in Figure 17.

The obtained results suggest that the use of metallized polyethylene as a basis for the creation of phase transition heat accumulators is promising. The limit temperatures that were achieved during heating in comparison with non-metallized PE are higher by 10–40 °C. The time needed to reach the limit temperature, which was achieved for pure polyethylene (164 °C), in the case of the copper content of 7 wt.% is reduced from 150 to 103 min, and the increase in the metal content to 15 wt.% affects an even more significant reduction in the charge time (90 min). The obtained values of temperature and time of charge and discharge of the heat accumulator model indicate the higher thermal conductivity of the obtained metal-filled system. As noted earlier, the higher thermal conductivity of polyethylene-based heat storage materials is a prerequisite for creating highly efficient heat accumulators.

**Figure 18:**
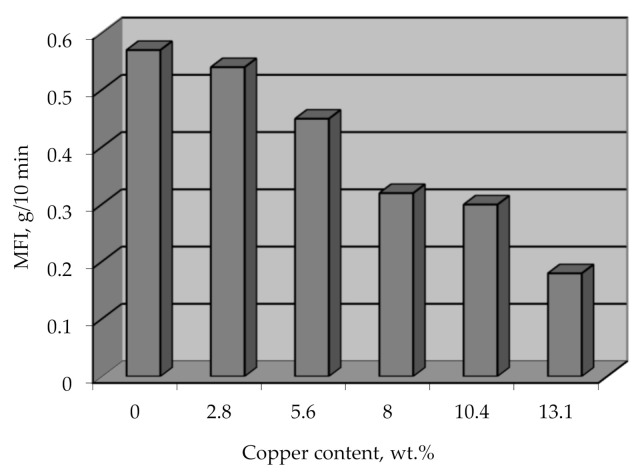
MFI vs copper content on metallized polyethylene granules.

The option of processing metallized polyethylene granules by conventional methods, which will allow obtaining metal-filled products directly during the processing of this raw material, should not be ruled out. This method of obtaining metal-filled products is promising because it eliminates a separate stage of mixing metal with polymer. The mixing occurs at elevated temperatures and at high shear rates, which causes the destruction of the polymer and affects the physical and mechanical properties of such composites. The effect of the metal on the viscosity of the polymer composite was determined by the melt flow index (MFI), and it was found that the decrease in MFI for a metal content of 13 wt.% is 30% (Figure 18). This information can be used to select the base grade of the polymer intended for metallization to obtain the desired value of MFI, depending on the chosen method of processing the material into products.

The MFI reduction with increasing the metal amount may be caused by different thicknesses of the copper coating on the polyethylene granules, which will get ruined in different ways during the melting of the composite. A thin coating will disintegrate easier and into finer particles, while a thick coating will form comparatively large metal particles, creating a higher resistance for the melted flow.

## 4. Conclusions

Thus, the study of the zinc-activated polyethylene surface obtained by joint treatment of the polyethylene granules with fine zinc powder in a ball mill showed that this method can produce a polymer surface with catalytic properties, which allows its chemical metallization due to copper ion reduction. The weight of metal that can be obtained on the polymer surface by the developed technology can be changed by the number of remetallization cycles and allows obtaining raw polymer materials with a high metal content. The study of the kinetics of copper ion reduction revealed that the rate of metallization of polyethylene granules depends on the history of the granules used and is determined by the surface area in contact with the chemical reducing solution, which decreases due to the smoothing of the micro-irregularities during subsequent remetallization cycles. The amount of copper that can be obtained on polyethylene granules is high and approaches the theoretical values.

The obtained preliminary results suggest that using metallized polyethylene as a basis for the creation of heat storage systems offers good prospects. In the case of metallized polyethylene, the time of charging and discharging the accumulator is much shorter compared to the initial polyethylene, which indicates an increased thermal conductivity of the metal-filled polymer system.

## Data Availability

The data presented in this study are available on request from the corresponding author.
